# Cognitive-behavioural pathways from pain to poor sleep quality and emotional distress in the general population: The indirect effect of sleep-related anxiety and sleep hygiene

**DOI:** 10.1371/journal.pone.0260614

**Published:** 2022-01-21

**Authors:** Arman Rakhimov, Daniel Whibley, Nicole K. Y. Tang

**Affiliations:** 1 Department of Psychology, University of Warwick, Coventry, West Midlands, United Kingdom; 2 Department of Physical Medicine and Rehabilitation, University of Michigan, Ann Arbor, Michigan, United States of America; 3 Epidemiology Group, School of Medicine, Medical Sciences and Nutrition, University of Aberdeen, Aberdeen, Scotland, United Kingdom; Swinburne University of Technology Faculty of Health Arts and Design, AUSTRALIA

## Abstract

**Objectives:**

Pain can have a negative impact on sleep and emotional well-being. This study investigated whether this may be partly explained by maladaptive sleep-related cognitive and behavioural responses to pain, including heightened anxiety about sleep and suboptimal sleep hygiene.

**Methods:**

This cross-sectional study used data from an online survey that collected information about pain (Brief Pain Inventory), sleep (Pittsburgh Sleep Quality Index; Sleep Hygiene Index; Anxiety and Preoccupation about Sleep Questionnaire) and emotional distress (PROMIS measures; Perceived Stress Scale). Structural equation modelling examined the tenability of a framework linking these factors.

**Results:**

Of 468 survey respondents (mean age 39 years, 60% female), 29% reported pain (mean severity 1.12), most commonly in the spine or low back (28%). Pain severity correlated with poor sleep quality, poor sleep hygiene, anxiety about sleep and emotional distress. In the first structural equation model, indirect effects were identified between pain severity and sleep quality through anxiety about sleep (β = .08, *p* < .001) and sleep hygiene (β = .12, *p* < .001). In the second model, an indirect effect was identified between pain severity and emotional distress through sleep quality (β = .19, *p* < .01). Combining these models, indirect effects were identified between pain severity and emotional distress through anxiety about sleep, sleep hygiene and sleep quality.

**Conclusions:**

This study provides data to support the tenability of a theoretically guided framework linking pain, sleep and emotional distress. If upheld by experimental and/or longitudinal study, this framework holds the potential to inform public health initiatives and more comprehensive pain assessment.

## Introduction

Pain is a leading cause of disability worldwide and its prevalence is increasing in aging populations [1]. It presents a substantial burden to the individual, healthcare services and society and can impact negatively on both sleep [2, 3] and mental health [4]. If left untreated, pain-related sleep disruption may become chronic, causing emotional distress, with the potential for the development of psychopathology [5]. Improved understanding of how pain impacts on sleep and mental health is therefore important to ensure interventions that aim to reduce or prevent pain-related poor health outcomes target the most relevant contributing factors.

Studies into the pain-sleep association have largely focused on the direction and the strength of the relationship [6]. Although evidence indicates that sleep is a stronger driver of pain, a bidirectional association is accepted and studies of adult populations have shown that pain can have a negative, downstream impact on sleep quality and insomnia symptom onset [2, 7]. As understanding of the association has improved, attention has shifted to the role of biological, psychosocial, and behavioural factors on pathways between pain and sleep. However, the majority of studies have conceptualised pathways from sleep variables to pain rather than *vice versa* [8]. Furthermore, sleep-specific psychological and/or behavioural factors, including sleep-related anxiety and sleep hygiene behaviours, are yet to be examined as possible mediators.

Both anxiety about sleep and sleep hygiene behaviours may be influenced by pain and may contribute to sleep quality. Previous research conducted with people with chronic pain has demonstrated associations between pain intensity and specific sleep hygiene behaviours (e.g. napping [9] and atypical bedtimes [10]). However, a lack of a correlation between pain intensity and sleep hygiene, captured by the Sleep Hygiene Index, has led to the proposition of indirect paths between pain intensity and sleep hygiene, including via anxiety [11]. Both anxiety about sleep and sleep hygiene have been associated with poor sleep quality [11–13] and poor sleep hygiene has been associated with the presence and persistence of insomnia [14]. An examination of the contribution of anxiety about sleep and sleep hygiene on paths between pain severity, sleep quality and emotional distress could provide empirical evidence to guide development of an expanded framework to elucidate the complexity of the pain-sleep relationship and to support applications of cognitive-behavioural interventions for insomnia comorbid with pain. For example, heightened anxiety about sleep and suboptimal sleep hygiene behaviours could represent maladaptive responses to pain and, if addressed early, could diminish sleep quality deterioration, breaking a chain to emotional distress.

Developing models that consider the impact of the pain-sleep association on mental health is particularly pertinent given that, like pain, anxiety and major depressive disorder are leading global causes of disability [1]. To incorporate greater complexity, modelling techniques that simultaneously estimate multiple effects between pain, sleep and mental health variables are needed. Structural Equation Modelling (SEM) can be applied to this end. SEM incorporates factor and path analyses to estimate direct and indirect effects between theoretically associated variables [15], while also addressing imprecision inherent in subjective measurements of pain, sleep quality and emotional distress. Accordingly, the aim of this study was to delineate a model that described direct and indirect pathways between pain severity, sleep-related factors and indicators of emotional distress in the general population. It was hypothesized that the association between pain severity and sleep quality would be partly explained by sleep-related anxiety and sleep hygiene, and that the association between pain severity and emotional distress would be explained, in part, by the effect of sleep-related cognitive-behavioural factors on sleep quality.

## Materials and methods

### Procedure

Participants completed an online survey hosted via Qualtrics (www.qualtrics.com), a widely used and participant-friendly platform, which participants could securely access via a link in a recruitment advertisement after providing online informed consent. The study protocol was approved by the Humanities and Social Sciences Research Ethics Committee of the University of Warwick, UK, (approval number PGR_18-19/18) with participants recruited between May and July 2019.

### Participants

A self-selected convenience sample was recruited using a university research participant recruitment platform, a university database of older adults interested in participating in research, and through advertisement on Facebook. To be eligible, participants had to be ≥18 years old and able to complete an online survey in the English language. On survey completion, participants were given a £5 eGift Voucher.

### Measures

The survey included questions about demographic and health-related information, pain, sleep-related factors, and dimensions of emotional well-being.

#### Demographic and health-related factors

Participants answered closed-ended questions about their age, gender, highest educational qualification, employment status, marital status, and pregnancy status if applicable (“Are you currently expecting a baby in the next 12 months?”). They were also asked about their perceived health status (“In general would you say your health is excellent, very good, good, fair, or poor?”), any history of sleep disorders (“Have you ever been diagnosed as having a sleep disorder, e.g. sleep apnoea, restless leg syndrome, narcolepsy?”), and use of sleep modifying medications (“Are you currently using any sleep medication?”) or other medications that can affect sleep (“Are you currently using any other medication, prescribed or not prescribed, that may affect your sleep?”).

#### Pain

Pain was assessed using the 9-item Brief Pain Inventory Short Form (BPI-SF) [16], an IMMPACT recommended core outcome measure for pain [17]. This instrument asks about pain severity and pain interference in the last 24 hours, with possible responses on two eleven-point scales ranging from 0 (no pain) to 10 (pain as bad as you can imagine) (pain severity subscale) and 0 (does not interfere) to 10 (completely Interferes) (pain interference subscale). This instrument has demonstrated good criterion and content validity as well as good test-retest reliability and internal consistency [18–21]. Pain location was assessed using a body manikin divided into areas based on the Manchester definition of widespread pain [22]. Respondents were asked to indicate with an X the region of the body that “hurts the most”.

#### Sleep quality

The Pittsburgh Sleep Quality Index (PSQI) was used to asses sleep quality [23]. The PSQI asks about 7 components of sleep over the last month (sleep latency, sleep duration, habitual sleep efficiency, sleep disturbances, subjective sleep quality, use of sleeping medication, and daytime dysfunction). Component scores are summed to calculate a global score (possible range 0–21, higher scores indicating poorer sleep quality). A global score >5 can be used to distinguish poor sleepers from good sleepers [23]. The PSQI has been shown to have good content validity, test-retest reliability and a high level of internal consistency [23–26].

#### Sleep hygiene

The Sleep Hygiene Index (SHI) consists of 13 items that assess practice of sleep hygiene behaviours [27]. Respondents are asked to assign a rating on a 5-point scale (0 “never”, 4 “always”) in response to the truth of each statement for them (e.g., “I go to bed at different times from day to day”). A total score (possible range 0–52) is obtained by summing all ratings, with higher scores representing poorer sleep hygiene. This instrument has been reported as having good construct validity and test–retest reliability [27].

#### Anxiety about sleep

The Anxiety and Preoccupation about Sleep Questionnaire (APSQ) [13], assesses anxiety about sleep problems. The instrument contains 10 items (e.g. “I worry about the amount of sleep I am going to get every night”), with respondents asked to rate the truth of each statement for them over the past month (1 “not true”; 10 “very true”). Higher summed scores (possible range 10–100) represent more anxiety and preoccupation about sleep. The APSQ has been shown to have good discriminant validity, convergent validity and internal consistency [13, 28].

#### Symptoms of anxiety and depression

Symptoms of anxiety and depression were measured with the Patient-Reported Outcomes Measurement Information System (PROMIS) Emotional Distress-Anxiety Short Form and the PROMIS Emotional Distress-Depression Short Form, respectively [29]. Respondents are asked to assess their feelings/symptoms over the past week (e.g., “My worries overwhelmed me” in the anxiety scale; “I felt hopeless” in the depression scale). Each response scale consists of four items, rated 1 “Never” to 5 “Always”, yielding a possible maximum raw score of 20. Higher scores indicate higher levels of anxiety/depression. These instruments have demonstrated good construct validity [29].

#### Stress

The Perceived Stress Scale (PSS) is a 10-item questionnaire used to measure stress level in the past month [30], e.g., “In the last month, how often have you found that you could not cope with all the things that you had to do?” Item scores range from 0 (never) to 4 (very often), with a summed possible total score range of 0–40 (higher score indicating higher levels of perceived stress). The scale has high concurrent and predictive validity, and adequate internal and test-retest reliability [30].

### Data analysis

Frequencies and percentages were calculated for all categorical variables and means and standard deviations (SD) for continuous variables, both for the total sample and for subgroups with and without pain. Inclusion in the pain subgroup was determined by a positive response to question 1 of the BPI-SF: ‘“Throughout our lives, most of us have had pain from time to time (such as minor headaches, sprains, and toothaches). Have you had pain other than these everyday kinds of pain today?”. To determine statistically significant differences between pain status subgroups, Chi-square tests of independence (for categorical variables), two-tailed t-tests of independence and Welch t-test (for continuous variables) were performed. To determine effect size, V-Cramer’s for categorical variables and d-Cohen’s for continuous variables were calculated. Associations between all variables were determined using Pearson correlational analyses. A *p* value < .05 was used to determine statistical significance, and Cohen’s guidelines were used to interpret correlation strength (weak: 0.1<*r*<0.3.; medium: 0.3<*r*<0.5; strong: *r*>0.5) [31]. All respondents were included in correlational analyses and structural equation modelling. Structural equation modelling with maximum likelihood estimation (IBM SPSS AMOS statistical package, version 26) was then used to examine direct and indirect paths between pain, sleep and emotional distress variables. A bootstrapping method with 5,000 bootstrap resamples and 95% bias-corrected confidence intervals was used. Analyses were carried out in two steps [32]. In step one, a measurement model was tested in which each factor could covary with every other factor. Multicollinearity was addressed by removing factors if correlation exceeded r = ±80 [32]. As some latent variables were comprised of multiple items there was a possibility of inflated measurement error. To mitigate this, item parcelling was performed with three parcels constructed as per recommendations [33]. We followed an algorithm described by Russel [34]: we fit a one-factor model to the items, rank-ordered them on the basis of their loadings and summed them together so that the average loadings of each group of items on the factor were as equal as possible. For the PSQI, summed subscales were used instead of items. If the measurement model provided an acceptable fit to the data, in step two iterative structural models were developed. Initially, a model that specified anxiety about sleep and sleep hygiene on the path between pain severity and sleep quality was examined (Model 1); then sleep quality on the path between pain severity and emotional distress was examined (Model 2); and finally Models 1 and 2 were combined in a composite model (Model 3). Models were evaluated using several fit indices: normed-fit index (NFI), comparative fit index (CFI), Tucker–Lewis index (TLI) and root-mean-square error of approximation (RMSEA). TLI, CFI and NFI values ≥ .95 indicated good model fit; values ≥.90 indicated acceptable model fit. RMSEA values ≤ .05 indicated good model fit; values ≤ .08 were considered acceptable [35, 36]. Gaskin’s plugin was used to calculate specific indirect effects [37].

## Results

Five-hundred individuals completed the online survey, with 468 participants included in analysis (188 males; 278 females; 2 people who identified as non-binary; mean age 39.0 years, SD = 19.4). Of the 500 who completed the survey, respondents were excluded due to missing data for demographic and health-related factors (n = 21), missing PSQI data (n = 2), and having more than 30% missing data (n = 1). Two participants who were pregnant were also excluded. Six multivariate outliers were also identified and excluded (>3 SD). In the resulting dataset (n = 468) a single imputation method was performed (i.e., mean/median substitution) [38], since there were <5% missing responses for each variable. Additionally, we compared results of correlational analyses from a dataset with imputed data to a dataset with observed values only (i.e., listwise deletion) and did not find meaningful differences. Therefore, the complete dataset with imputed values was used in further analyses.

### Internal consistency of measurement instruments

Internal consistency of all instruments was analysed in the current sample and found to be reasonable to excellent and comparable to previous reports: BPI-SF: Cronbach’s α = .86 (pain severity subscale) and Cronbach’s α = .91 (pain interference subscale); PSQI: Cronbach’s α = .76; PROMIS Emotional Distress-Anxiety Short Form: Cronbach’s α = .93; PROMIS Emotional Distress-Depression Short Form: Cronbach’s α = .95; PSS: Cronbach’s α = .88; SHI: Cronbach’s α = .83; and APSQ: Cronbach’s α = .95.

### Participant characteristics

Descriptive statistics for the sample are presented in Table 1. All participants were between 18 and 85 years (mean = 39, SD = 19.4). Most were female (60%) and of those who provided information about their ethnicity, 78% were white. The majority had no history of a diagnosed sleep disorder and did not take sleep or non-sleep medications. The proportion of good and poor sleepers was 52% (N = 244) and 48% (N = 224) respectively. Of the 28.6% (N = 134) who reported pain, on average, severity and interference were mild (mean = 1.11, SD = 2.02 and mean = 1.19, SD = 2.29, respectively). The most common location of pain (see S1 and S2 Tables) was the spine or low back (28%), followed by the head (19%), knee, lower leg, ankle or foot (17%), hip or thigh (13%), abdomen (8%), shoulder or upper back (7%), forearm, wrist or hand (2%), neck (1%) and chest (1%) (4% had missing data for pain location). Participants with and without pain did not differ in terms of ethnicity, education and employment status. When compared to individuals without pain, people with pain were significantly more likely to be older, female, partnered, report worse health, have a history of sleep disorders, and be more likely to be taking sleep and/or non-sleep mediations.

**Table 1:** Participant characteristics and descriptive statistics of the study variables.

	Total	No pain	Pain	*χ*^2^/*t*	*p*	*V/d*
	*N* = 468	*n* = 334	*n* = 134			
Gender						
Female	278 (59.4%)	186 (55.7%)	92 (68.7%)	6.79	.021	.12
Male	188 (40.2%)	146 (43.7%)	42 (31.3%)			
Non-binary	2 (0.4%)	2 (0.6%)	0 (0.0%)			
Age (*M*, SD)	38.97 ± 19.40	36.84 ± 18.76	44.30 ± 20.00	-3.81	< .001	.39
Ethnicity						
Asian	76 (16.2%)	54 (16.2%)	22 (16.4%)	1.83	.801	.06
Black	12 (2.6%)	10 (3.0%)	2 (1.5%)			
Mixed	11 (2.4%)	8 (2.4%)	3 (2.2%)			
White	365 (78.0%)	260 (77.8%)	105 (78.4%)			
Other	4 (0.9%)	2 (0.6%)	2 (1.5%)			
Educational level						
University degree	269 (57.5%)	185 (55.4%)	84 (62.7%)	2.08	.179	.07
No university degree	199 (42.5%)	149 (44.6%)	50 (37.3%)			
Employment status						
Employed/Self-employed	176 (37.6%)	135 (40.4%)	41 (30.6%)	3.93	.057	.09
Other (studying, etc.)	292 (62.4%)	199 (59.6%)	93 (69.4%)			
Marital status						
Married/In partnership	204 (43.6%)	133 (39.8%)	71 (53.0%)	6.74	.010	.12
Single	264 (56.4%)	201 (60.2%)	63 (47.0%)			
Perceived health status (*M*, SD)	2.71 ± 1.00	2.54 ± 0.91	3.14 ± 1.08	-6.13	< .001	.60
Good health	366 (78.2%)	280 (83.8%)	86 (64.2%)			
Poor health	102 (21.8%)	54 (16.2%)	48 (35.8%)			
History of sleep disorders						
Yes	34 (7.3%)	14 (4.2%)	20 (14.9%)	16.35	< .001	.19
No	434 (92.7%)	320 (95.8%)	114 (85.1%)			
Taking sleep medication						
Yes	24 (5.1%)	9 (2.7%)	15 (11.2%)	14.20	.001	.17
No	444 (94.9%)	325 (97.3%)	119 (88.8%)			
Taking non-sleep medication						
Yes	55 (11.8%)	28 (8.4%)	27 (20.1%)	12.77	.001	.17
No	413 (88.2%)	306 (91.6%)	107 (79.9%)			
Pain interference	1.19 ± 2.29	0.00 ± 0.00	4.15 ± 2.45	-	-	-
Pain severity	1.11 ± 2.02	0.00 ± 0.00	3.88 ± 1.85	-	-	-
Sleep quality	6.17 ± 3.51	5.40 ± 2.98	8.08 ± 3.99	-7.03	< .001	.81
Depression	52.85 ± 11.29	50.90 ± 10.80	57.80 ± 11.00	-6.24	< .001	.64
Anxiety	54.41 ± 11.30	52.40 ± 11.10	59.40 ± 10.20	-6.52	< .001	.64
Perceived stress	17.99 ± 7.89	17.00 ± 8.00	21.00 ± 8.00	-5.01	< .001	.51
Sleep hygiene	17.24 ± 8.36	17.00 ± 8.00	19.00 ± 9.00	-2.79	.006	.29
Anxiety about sleep	42.32 ± 23.22	39.29 ± 22.26	49.87 ± 23.92	-4.55	< .001	.47

Participants, who answered “Yes” to the first question of the BPI-SF, “Throughout our lives, most of us have had pain from time to time (such as minor headaches, sprains, and toothaches). Have you had pain other than these everyday kinds of pain today?” were included in the “Pain” group. Participants who answered “No” were included in the “No pain” group. Chi-square test of independence was conducted with categorical variables. SPSS Statistics’ Exact Module was employed where cells have expected count less than 5: gender and ethnicity. Two-tailed t-test of independence and Welch t-test were conducted with continuous variables. V-Cramer’s effect size was calculated for categorical variables. d-Cohen’s effect size was calculated for continuous variables.

### Correlational analysis

Table 2 presents the results of correlational analyses. Pain severity was moderately and positively correlated with poor sleep quality, anxiety and depression. Pain severity was also positively correlated with poor sleep hygiene, anxiety about sleep (both weak correlations) and perceived stress (close to the pre-defined threshold for moderate correlation).

**Table 2:** Pearson correlations between variables used in the structural equation models.

	1.	2.	3.	4.	5.	6.
1. Pain severity	—					
2. Sleep quality	.44	—				
3. Sleep hygiene	.16	.41	—			
4. Anxiety about sleep	.25	.55	.57	—		
5. Anxiety	.31	.51	.60	.61	—	
6. Depression	.32	.53	.55	.54	.86	—
7. Perceived stress	.29	.48	.56	.57	.81	.80

N = 468. For sleep quality and sleep hygiene higher scores indicate poorer sleep quality/sleep hygiene. For all correlations, p < .001.

### Structural equation modeling

#### Measurement model

The measurement model was comprised of five latent factors: pain severity, anxiety and preoccupation about sleep, sleep hygiene, sleep quality and emotional distress. The pain severity factor was measured by four indicator variables (individual items on the pain severity subscale of the BPI-SF). Anxiety about sleep, sleep hygiene and sleep quality factors were comprised of three parcels each. The emotional distress factor was measured by global scores from the Perceived Stress Scale, PROMIS Anxiety Scale Short Form and PROMIS Depression Scale Short Form. The model was adjusted for age, gender, ethnicity, employment, marital status, educational level, perceived health, sleep medication use, non-sleep medication use, and history of sleep disorders. The model had acceptable fit statistics (*χ2* = 544.43, df = 204, *χ2/df* = 2.67, *p* < .001, NFI = .93; CFI = .96; TLI = .93; RMSEA = .06 (90% CI: .05-.07). The standardized factor loadings were substantial, ranging from .69 to .96, *Mdn* = .89, *p* ≤ .001. Correlations between latent factors were statistically significant (*p* < .001).

#### Structural models

**Fig 1:**
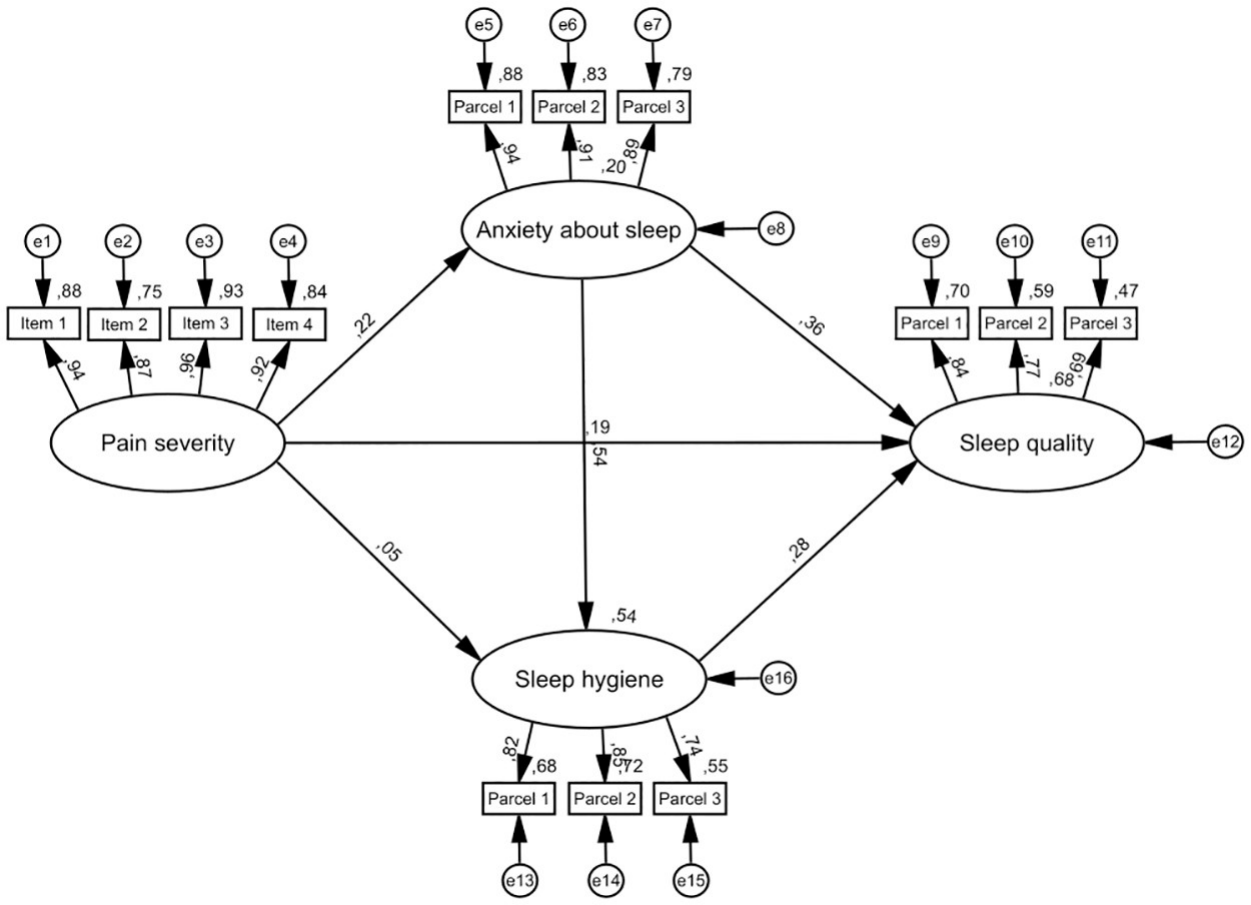
Model 1—Indirect paths between pain severity and sleep quality. *N* = 468. Rectangles represent observed variables and ovals represent latent variables. All values are standardized coefficients, except for values near variables, which are squared multiple correlations. Error terms of variables are depicted as e1-e12. Control variables (age, gender, ethnicity, employment and marital status, educational level, perceived health, taking sleep medications, taking non-sleep medications and history of sleep disorders) are not depicted to support visual clarity. For sleep quality and sleep hygiene higher scores indicate poorer sleep quality/sleep hygiene.

**Fig 2:**
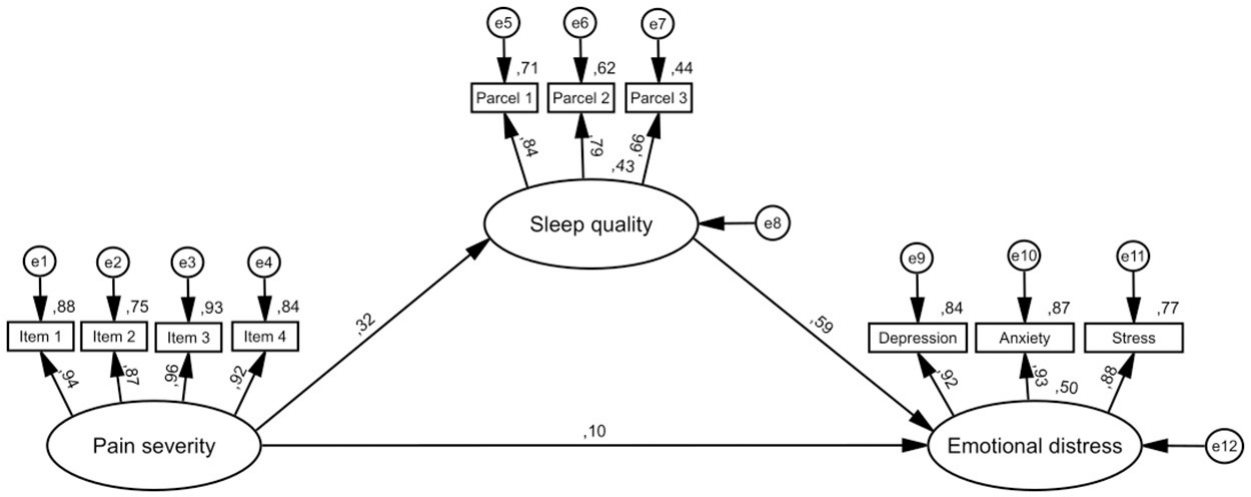
Model 2—Sleep quality on an indirect path between pain severity and emotional distress. N = 468. Rectangles represent observed variables and ovals represent latent variables. All values are standardized coefficients, except for values near variables, which are squared multiple correlations. Error terms of variables are depicted as e1-e16. Control variables (age, gender, ethnicity, employment and marital status, educational level, perceived health, taking sleep medications, taking non-sleep medications and history of sleep disorders) are not depicted to support visual clarity. For sleep quality and sleep hygiene higher scores indicate poorer sleep quality/sleep hygiene.

**Fig 3:**
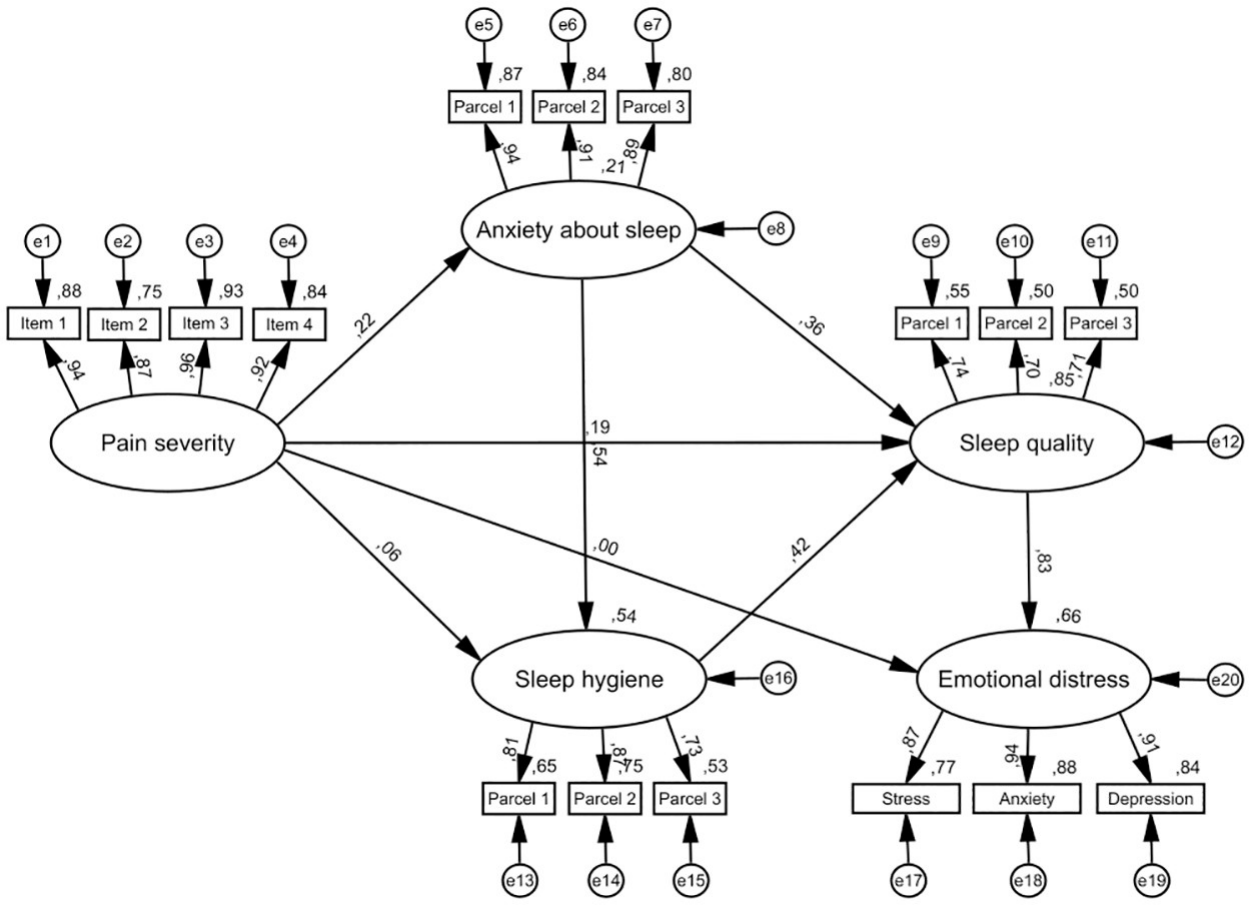
Model 3—Pathways linking pain severity, sleep quality and emotional distress. N = 468. Rectangles represent observed variables and ovals represent latent variables. All values are standardized coefficients, except for values near variables, which are squared multiple correlations. Error terms of variables are depicted as e1-e20. Control variables (age, gender, ethnicity, employment and marital status, educational level, perceived health, taking sleep medications, taking non-sleep medications and history of sleep disorders) are not depicted to support visual clarity. For sleep quality and sleep hygiene higher scores indicate poorer sleep quality/sleep hygiene.

Figs 1–3 depict iterative, pre-specified structural models. Table 3 presents direct and indirect effects from all models.

**Table 3:** Standardized direct and indirect effects for models 1–3.

	Model 1		Model 2		Model 3	
	Direct effect	Indirect effect	Direct effect	Indirect effect	Direct effect	Indirect effect
**Pain severity**						
→Sleep quality	.19 ^b^		.32 ^b^		.19 ^b^	
→Anxiety about sleep	.22^a^				.22 ^b^	
→Sleep hygiene	.05				.06	
→Emotional distress			.10		.00	
→Anxiety about sleep→Sleep quality		.08 ^b^				.08 ^b^
→Anxiety about sleep→Sleep hygiene→Sleep quality		.12 ^b^				.12 ^a^
→Anxiety about sleep→Sleep quality→Emotional distress						.08 ^b^
→Anxiety about sleep→Sleep hygiene→Sleep quality→Emotional distress						.12 ^a^
→Sleep hygiene→Sleep quality		.02				.02
→Sleep hygiene→Sleep quality→Emotional distress						.02
→Sleep quality→Emotional distress				.19 ^a^		.16 ^a^
**Anxiety about sleep**						
→Sleep quality	.36 ^b^				.36 ^b^	
→Sleep hygiene	.54 ^a^				.54 ^a^	
**Sleep hygiene**						
→Sleep quality	.28 ^a^				.42 ^a^	
**Sleep quality**						
→Emotional distress			.59 ^a^		.83 ^a^	

N = 468. Model 1—direct and indirect effects of pain severity on sleep quality. Model 2—direct and indirect effects of pain severity on emotional distress. Model 3—direct and indirect effects of pain severity on sleep quality and emotional distress. For sleep quality and sleep hygiene higher scores indicate poorer sleep quality/sleep hygiene.

^a^ *p* < .01,

^b^ *p* < .001.

Model 1 (Fig 1) tested the direct effect of pain severity on sleep quality as well as indirect effects through two parallel indirect paths (anxiety about sleep, and sleep hygiene). Modification indices in SPSS AMOS suggested an additional path from anxiety about sleep to sleep hygiene. Given previous proposition that anxiety may lie on an indirect path between pain intensity and sleep hygiene [11], this additional path was added to the model. This addition improved overall model fit (*χ2* = 381.30, df = 149, *χ2/df* = 2.56, *p* < .001, NFI = .94; CFI = .96; TLI = .93; RMSEA = .06 (90% CI: .05-.07)). The direct effect of pain severity on sleep quality was statistically significant (β = .19, *p* < .001), as was the indirect effect of pain severity on sleep quality through anxiety about sleep (β = .08, *p* < .001), and the indirect effect of pain severity on sleep quality through anxiety about sleep and sleep hygiene (β = .12, *p* < .001). However, the indirect effect of pain severity on sleep quality through sleep hygiene alone was not statistically significant (β = .02, *p*>.05). This model accounted for 68% of the total variance of sleep quality.

Model 2 (Fig 2) specified sleep quality on the indirect path between pain severity and emotional distress. Fit indices were acceptable: *χ2* = 276.55, df = 103, *χ2/df* = 2.69, *p* < .001, NFI = .95; CFI = .97; TLI = .94; RMSEA = .06 (90% CI: .05-.07). The direct effect of pain severity on emotional distress was not statistically significant (β = .10, *p*>.05). However, the indirect effect of pain severity on emotional distress via sleep quality was (β = .19, *p* < .01). This model accounted for 50% of the total variance of emotional distress.

Model 3 (Fig 3) was identical to model 1, except that the emotional distress latent variable was added as well as two additional paths: from pain severity and from sleep quality to emotional distress. The model demonstrated acceptable fit to the data: *χ2* = 603.08, df = 207, *χ2/df* = 2.91, *p* < .001, NFI = .92; CFI = .95; TLI = .92; RMSEA = .06 (90% CI: .06-.07). Pain severity was associated with sleep quality (β = .19, *p* < .001), and anxiety about sleep (β = .22, *p* < .001), but not with sleep hygiene (β = .06, *p* > .05), or emotional distress (β = .00, *p* > .05). Two indirect paths connecting pain severity and sleep quality were statistically significant: through anxiety about sleep (β = .08, *p* < .001), and two sequential factors: anxiety about sleep and sleep hygiene (β = .12, *p* = < .01). The indirect path between pain severity and sleep quality through sleep hygiene was not statistically significant (β = .02, *p*>.05). Three indirect paths connecting pain severity and emotional distress were statistically significant. The first path involved sleep quality as a mediator (β = .16, *p* < .01). The second path involved two mediators: anxiety about sleep and then sleep quality (β = .08, *p* < .001). The third path involved three mediators: anxiety about sleep, then sleep hygiene and then sleep quality (β = .12, *p* < .01). The indirect path connecting pain severity and emotional distress (via sleep hygiene and sleep quality) was not statistically significant (β = .02, *p*>.05). Overall, this model accounted for 85% of the total variance of sleep quality and 66% of the total variance of emotional distress.

## Discussion

The aim of this study was to develop a framework to describe associations between pain severity, sleep quality and emotional distress that incorporated sleep-related cognitions and behaviours. Through iterative model building, a framework is described within which pain and emotional distress are linked by indirect paths through anxiety about sleep, sleep hygiene behaviours and sleep quality. This model maps onto current theories and evidence linking pain severity, sleep-related factors and emotional distress [39]. With replication and further development, there is potential to inform public health messages about sleep-related responses to pain (i.e., reducing anxiety about sleep and maintaining positive sleep hygiene behaviours) to prevent reduction in sleep quality and emotional well-being. Sleep-related treatment targets for pain are also suggested, which may attenuate downstream effects on emotional well-being.

In the first model, an indirect pathway from pain severity to sleep quality through sleep-related anxiety was significant but a pathway through sleep hygiene was not. However, a path from pain to sleep-related anxiety to sleep hygiene to sleep quality *was* supported. As a cross-sectional study, causal inferences cannot be made. However, this exploratory finding warrants experimental and longitudinal examination. In the second model, the association between pain severity and emotional distress was mediated through sleep quality. Again, this is worthy of further investigation as the impact of pain on sleep quality and subsequent psychopathology has important translational ramifications. For example, associations of pain severity or chronic pain with Major Depressive Disorder and depressive symptoms [40, 41], anxiety and mood disorders [42], emotional distress [43, 44], and Post Traumatic Stress Disorder [45] may partly be explained by the effect of pain on sleep quality. The results of the current study are also coherent with models that posit sleep optimization as a key facet of preventative medicine, guarding against poor mental health [46].

Of the scarce studies that have examined mediators on the path from pain to sleep, both have focused on specific conditions. Among adults with rheumatoid arthritis, the association between pain intensity and sleep disturbance (measured using the PSQI) was partly accounted for by an indirect pathway through symptoms of depression [47]. In a daily diary study with children with Sickle Cell Disease, negative mood (assessed using the Facial Affective Scale) partly explained the association between pain intensity and subjective sleep quality [48]. We believe this is the first study to examine sleep-related anxiety and sleep hygiene on the path between pain and sleep quality, and findings demonstrate the value in collecting information about these sleep-specific constructs as part of a routine pain assessment.

With respect to the role of sleep-related factors in the association between pain and emotional distress, previous examinations applying path analysis are restricted to study of people with fibromyalgia. Miro *et al*. reported on the significance of subjective sleep quality on an indirect path between pain intensity and emotional distress [49], later corroborated by Diaz-Piedra and colleagues, who extended the evidence by demonstrating a comparable path through polysomnography-measured sleep efficiency [50]. Again, our findings suggest that collecting data on sleep-specific cognitions and behaviours may expand understanding of underlying mechanisms, and we recommend future longitudinal examinations within both general and condition-specific populations. If supported by prospective observational research and randomized trials with embedded mediation analyses, a case would be strengthened for the effectiveness of Cognitive Behavioural Therapy for Insomnia (CBT-I) as preventative for psychopathology for those presenting with pain in primary care. This would also provide additional evidence for incorporation of CBT-I into pain management for those with chronic pain and comorbid insomnia [39].

Study strengths include use of measurement instruments with good psychometric properties and the use of structural equation modelling to address measurement error and to create latent variables to better represent the constructs of interest. The study had an adequate sample size to undertake the pre-specified analysis, and robustly tested our hypotheses through an iterative model building process. Convenience sampling was used as it is a cost-effective approach to recruit participants from the general population, and recruitment of participants from the general population provided a better representation than would have been achieved by stipulating condition-specific exclusion criteria. However, this sampling strategy resulted in recruitment of people with, on average, pain of mild severity. Study limitations include the cross-sectional design and, accordingly, no causal claims are made; the hypothesised directionality must be tested in experimental and longitudinal studies. However, models in the present study provide a template to inform future data collection. Although there was adjustment for a large number of covariates, residual confounding should also be addressed in future study. For example, hypervigilance or attention to pain is associated with pain reporting/pain severity [51], sleep disturbance [52], and negative emotions [53]. Pain self-efficacy is also likely to be important as it has been reported as on the indirect path between pain intensity and dimensions of emotional distress among people with chronic pain [49]. In this study pain was conceptualised as a presenting symptom and limited information about the pain source was collected. However, we believe that our general population approach offers a generic framework that can adapted for use in the future study of specific conditions. Similarly, we did not assign participants to an insomnia disorder group, an insomnia symptoms group, or a ‘good sleeper’ group as has been previously undertaken [54]. More comprehensive phenotyping in future studies would help to better understand the importance of such categorisation. In addition, future studies should confirm the validity of the proposed framework, including examining its tenability in age- and sex-stratified analyses given sleep-related cognitions and behaviours may differ according to different demographics. An additional limitation is the self-reported nature of all variables under study. However, given their multidimensional nature, future research that uses objective measures would broaden our understanding, rather than replace or validate the subjective experiences that we have captured.

In conclusion, this study examined associations between pain severity, sleep-related anxiety, sleep hygiene behaviour, sleep quality and emotional distress and proposed an empirically driven and theoretically guided framework. Confirmation of this model using prospectively collected and experimental data would are required to substantiate any potential causality, as well as to inform and diversify public health initiatives for promoting well-being in the general population.

## Supporting information

S1 Table Body map area. (DOCX)

S2 Table Pain locations. (DOCX)

## References

[1] Vos T, Abajobir AA, Abate KH, Abbafati C, Abbas KM, Abd-Allah F, et al. Global, regional, and national incidence, prevalence, and years lived with disability for 328 diseases and injuries for 195 countries, 1990–2016: a systematic analysis for the Global Burden of Disease Study 2016. The Lancet. 2017 Sep 16;390(10100):1211–59. 10.1016/S0140-6736(17)32154-2 PMC5605509 28919117

[2] Jansson‐Fröjmark M, Boersma K. Bidirectionality between pain and insomnia symptoms: A prospective study. British Journal of Health Psychology. 2012;17(2):420–31. doi: 10.1111/j.2044-8287.2011.02045.x 22106955

[3] O’Brien EM, Waxenberg LB, Atchison JW, Gremillion HA, Staud RM, McCrae CS, et al. Intraindividual Variability in Daily Sleep and Pain Ratings Among Chronic Pain Patients: Bidirectional Association and the Role of Negative Mood. The Clinical Journal of Pain. 2011 Jun;27(5):425–33. doi: 10.1097/AJP.0b013e318208c8e4 21415723

[4] Gerrits MMJG, van Oppen P, van Marwijk HWJ, Penninx BWJH, van der Horst HE. Pain and the onset of depressive and anxiety disorders. PAIN. 2014 Jan;155(1):53–9. doi: 10.1016/j.pain.2013.09.005 24012953

[5] Medic G, Wille M, Hemels M. Short- and long-term health consequences of sleep disruption. NSS. 2017 May; 9:151–61. doi: 10.2147/NSS.S134864 28579842 PMC5449130

[6] Finan PH, Goodin BR, Smith MT. The Association of Sleep and Pain: An Update and a Path Forward. The Journal of Pain. 2013 Dec 1;14(12):1539–52. doi: 10.1016/j.jpain.2013.08.007 24290442 PMC4046588

[7] Amtmann D, Bamer AM, Askew R, Jensen MP. Cross-lagged longitudinal analysis of pain intensity and sleep disturbance. Disability and Health Journal. 2020 Jul 1;13(3):100908. doi: 10.1016/j.dhjo.2020.100908 32081590

[8] Whibley D, AlKandari N, Kristensen K, Barnish M, Rzewuska M, Druce KL, et al. Sleep and Pain. Clin J Pain. 2019 Jun;35(6):544–58. doi: 10.1097/AJP.0000000000000697 30829737 PMC6504189

[9] Theadom A, Cropley M, Kantermann T. Daytime napping associated with increased symptom severity in fibromyalgia syndrome. BMC Musculoskeletal Disorders. 2015 Feb 7;16(1):13. doi: 10.1186/s12891-015-0464-y 25888479 PMC4333241

[10] McHugh RK, Edwards RR, Ross EL, Jamison RN. Does bedtime matter among patients with chronic pain? A longitudinal comparison study. Pain Rep. 2019 May 9;4(3). doi: 10.1097/PR9.0000000000000747 31583360 PMC6749921

[11] Cho S, Kim G-S, Lee J-H. Psychometric evaluation of the sleep hygiene index: a sample of patients with chronic pain. Health Qual Life Outcomes. 2013 Dec 22;11(1):213. doi: 10.1186/1477-7525-11-213 24359272 PMC3905101

[12] Gellis LA, Lichstein KL. Sleep Hygiene Practices of Good and Poor Sleepers in the United States: An Internet-Based Study. Behavior Therapy. 2009 Mar 1;40(1):1–9. doi: 10.1016/j.beth.2008.02.001 19187812

[13] Tang NKY, Harvey AG. Correcting distorted perception of sleep in insomnia: a novel behavioural experiment? Behaviour Research and Therapy. 2004 Jan 1;42(1):27–39. doi: 10.1016/s0005-7967(03)00068-8 14744521

[14] Jansson-Fröjmark M, Evander J, Alfonsson S. Are sleep hygiene practices related to the incidence, persistence and remission of insomnia? Findings from a prospective community study. J Behav Med. 2019 Feb 1;42(1):128–38. doi: 10.1007/s10865-018-9949-0 29995266

[15] Amtmann D, Askew RL, Kim J, Chung H, Ehde DM, Bombardier CH, et al. Pain affects depression through anxiety, fatigue and sleep in Multiple Sclerosis. Rehabil Psychol. 2015 Feb;60(1):81–90. doi: 10.1037/rep0000027 25602361 PMC4349204

[16] Cleeland CS, Ryan KM. Pain assessment: global use of the Brief Pain Inventory. Ann Acad Med Singap. 1994 Mar;23(2):129–38. 8080219

[17] Dworkin RH, Turk DC, Farrar JT, Haythornthwaite JA, Jensen MP, Katz NP, et al. Core outcome measures for chronic pain clinical trials: IMMPACT recommendations. Pain. 2005 Jan;113(1–2):9–19. doi: 10.1016/j.pain.2004.09.012 15621359

[18] McDonald DD, Shea M, Fedo J, Rose L, Bacon K, Noble K, et al. Older Adult Pain Communication and the Brief Pain Inventory Short Form. Pain Management Nursing. 2008 Dec 1;9(4):154–159.e2. doi: 10.1016/j.pmn.2008.03.001 19041613

[19] Mendoza T, Mayne T, Rublee D, Cleeland C. Reliability and validity of a modified Brief Pain Inventory short form in patients with osteoarthritis. European Journal of Pain. 2006;10(4):353–353. doi: 10.1016/j.ejpain.2005.06.002 16051509

[20] Zalon ML. Comparison of pain measures in surgical patients. J Nurs Meas. 1999;7(2):135–52. 10710858

[21] Radbruch L, Loick G, Kiencke P, Lindena G, Sabatowski R, Grond S, et al. Validation of the German Version of the Brief Pain Inventory. Journal of Pain and Symptom Management. 1999 Sep 1;18(3):180–7. doi: 10.1016/s0885-3924(99)00064-0 10517039

[22] MacFarlane GJ, Croft PR, Schollum J, Silman AJ. Widespread pain: is an improved classification possible? J Rheumatol. 1996 Sep;23(9):1628–32. 8877936

[23] Buysse DJ, Reynolds CF, Monk TH, Berman SR, Kupfer DJ. The Pittsburgh sleep quality index: A new instrument for psychiatric practice and research. Psychiatry Research. 1989 May 1;28(2):193–213. doi: 10.1016/0165-1781(89)90047-4 2748771

[24] Backhaus J, Junghanns K, Broocks A, Riemann D, Hohagen F. Test–retest reliability and validity of the Pittsburgh Sleep Quality Index in primary insomnia. Journal of Psychosomatic Research. 2002 Sep;53(3):737–40. doi: 10.1016/s0022-3999(02)00330-6 12217446

[25] Carpenter JS, Andrykowski MA. Psychometric evaluation of the pittsburgh sleep quality index. Journal of Psychosomatic Research. 1998 Jul 1;45(1):5–13. doi: 10.1016/s0022-3999(97)00298-5 9720850

[26] de la Vega R, Tomé-Pires C, Solé E, Racine M, Castarlenas E, Jensen MP, et al. The Pittsburgh Sleep Quality Index: Validity and factor structure in young people. Psychol Assess. 2015 Dec;27(4):e22–27. doi: 10.1037/pas0000128 26653055

[27] Mastin DF, Bryson J, Corwyn R. Assessment of Sleep Hygiene Using the Sleep Hygiene Index. J Behav Med. 2006 Jun 1;29(3):223–7. doi: 10.1007/s10865-006-9047-6 16557353

[28] Jansson-Fröjmark M, Harvey AG, Lundh L-G, Norell-Clarke A, Linton SJ. Psychometric Properties of an Insomnia-Specific Measure of Worry: The Anxiety and Preoccupation about Sleep Questionnaire. Cognitive Behaviour Therapy. 2011 Mar 1;40(1):65–76. doi: 10.1080/16506073.2010.538432 21337216

[29] Cella D, Choi SW, Condon DM, Schalet B, Hays RD, Rothrock NE, et al. PROMIS^®^ Adult Health Profiles: Efficient Short-Form Measures of Seven Health Domains. Value in Health. 2019 May;22(5):537–44. doi: 10.1016/j.jval.2019.02.004 31104731 PMC7201383

[30] Cohen S, Kamarck T, Mermelstein R. A Global Measure of Perceived Stress. Journal of Health and Social Behavior. 1983;24(4):385–96. 6668417

[31] Cohen J. Statistical power analysis for the behavioral sciences. 2nd ed. Hillsdale, N.J: L. Erlbaum Associates; 1988.

[32] Hatcher L. Advanced Statistics in Research: Reading, Understanding, and Writing Up Data Analysis Results. Saginaw, MI: ShadowFinch Media, LLC; 2013.

[33] Matsunaga M. Item Parceling in Structural Equation Modeling: A Primer. Communication Methods and Measures. 2008 Dec 9;2(4):260–93.

[34] Russell DW, Kahn JH, Spoth R, Altmaier EM. Analyzing Data From Experimental Studies: A Latent Variable Structural Equation Modeling Approach. Journal of Counseling Psychology. 1998;45(1):18–29.

[35] Keith TZ. Multiple Regression and Beyond: An Introduction to Multiple Regression and Structural Equation Modeling. 2nd ed. New York: Routledge/Taylor & Francis Group; 2015.

[36] Schumacker RE, Lomax RG. A beginner’s guide to structural equation modeling. 3rd ed. New York, NY, US: Routledge/Taylor & Francis Group; 2010.

[37] Gaskin J. ‘Specific Indirect Effect’, Gaskination’s Statistics [Internet]. 2016. http://statwiki.gaskination.com/index.php?title=Plugins

[38] Hair JF, editor. Multivariate data analysis. 7th ed. Harlow: Pearson; 2014.

[39] Herrero Babiloni A, Beetz G, Tang NKY, Heinzer R, Nijs J, Martel MO, et al. Towards the endotyping of the sleep–pain interaction: a topical review on multitarget strategies based on phenotypic vulnerabilities and putative pathways. PAIN. 2021 May;162(5):1281–8. doi: 10.1097/j.pain.0000000000002124 33105436

[40] Ohayon MM. Specific Characteristics of the Pain/Depression Association in the General Population. J Clin Psychiatry. 2004 Aug 1;65(suppl 12):5–9. 15315471

[41] Patel KV, Cochrane BB, Turk DC, Bastian LA, Haskell SG, Woods NF, et al. Association of Pain With Physical Function, Depressive Symptoms, Fatigue, and Sleep Quality Among Veteran and non-Veteran Postmenopausal Women. The Gerontologist. 2016 Feb 1;56(Suppl_1):S91–101. 26768395 10.1093/geront/gnv670 PMC5881612

[42] McWilliams LA, Cox BJ, Enns MW. Mood and anxiety disorders associated with chronic pain: an examination in a nationally representative sample. PAIN. 2003 Nov;106(1):127–33. 14581119 10.1016/s0304-3959(03)00301-4

[43] Cai B, Oderda GM. The Association Between Pain and Depression and Some Determinants of Depression for the General Population of the United States. Journal of Pain & Palliative Care Pharmacotherapy. 2012 Sep 4;26(3):257–65. doi: 10.3109/15360288.2012.703292 22973915

[44] Walter SA, Jones MP, Talley NJ, Kjellström L, Nyhlin H, Andreasson AN, et al. Abdominal pain is associated with anxiety and depression scores in a sample of the general adult population with no signs of organic gastrointestinal disease. Neurogastroenterology & Motility. 2013;25(9):741–e576. 23692044 10.1111/nmo.12155

[45] Otis JD, Keane TM, Kerns RD. An examination of the relationship between chronic pain and post-traumatic stress disorder. 2003;40(5):9. 10.1682/jrrd.2003.09.0397 15080224

[46] Palagini L, Bastien CH, Marazziti D, Ellis JG, Riemann D. The key role of insomnia and sleep loss in the dysregulation of multiple systems involved in mood disorders: A proposed model. Journal of Sleep Research. 2019;28(6):e12841. doi: 10.1111/jsr.12841 30968511

[47] Nicassio PM, Ormseth SR, Kay M, Custodio M, Irwin MR, Olmstead R, et al. The contribution of pain and depression to self-reported sleep disturbance in patients with rheumatoid arthritis. PAIN. 2012 Jan;153(1):107–12. doi: 10.1016/j.pain.2011.09.024 22051047 PMC3245817

[48] Valrie CR, Gil KM, Redding-Lallinger R, Daeschner C. Brief Report: Daily Mood as a Mediator or Moderator of the Pain–Sleep Relationship in Children with Sickle Cell Disease. Journal of Pediatric Psychology. 2008 Apr 1;33(3):317–22. doi: 10.1093/jpepsy/jsm058 17690117

[49] Miró E, Martínez MP, Sánchez AI, Prados G, Medina A. When is pain related to emotional distress and daily functioning in fibromyalgia syndrome? The mediating roles of self-efficacy and sleep quality. British Journal of Health Psychology. 2011;16(4):799–814. doi: 10.1111/j.2044-8287.2011.02016.x 21988065

[50] Diaz-Piedra C, Catena A, Miro E, Martinez MP, Sanchez AI, Buela-Casal G. The Impact of Pain on Anxiety and Depression is Mediated by Objective and Subjective Sleep Characteristics in Fibromyalgia Patients. The Clinical Journal of Pain. 2014 Oct;30(10):852–9. doi: 10.1097/AJP.0000000000000040 24281292

[51] Herbert MS, Goodin BR, Pero ST IV, Schmidt JK, Sotolongo A, Bulls HW, et al. Pain Hypervigilance is Associated with Greater Clinical Pain Severity and Enhanced Experimental Pain Sensitivity Among Adults with Symptomatic Knee Osteoarthritis. Annals of Behavioral Medicine. 2014 Aug 1;48(1):50–60. doi: 10.1007/s12160-013-9563-x 24352850 PMC4063898

[52] Harrison L, Wilson S, Heron J, Stannard C, Munafò MR. Exploring the associations shared by mood, pain-related attention and pain outcomes related to sleep disturbance in a chronic pain sample. Psychol Health. 2016 May;31(5):565–77. doi: 10.1080/08870446.2015.1124106 26726076 PMC4966632

[53] Leeuw M, Goossens MEJB, Linton SJ, Crombez G, Boersma K, Vlaeyen JWS. The Fear-Avoidance Model of Musculoskeletal Pain: Current State of Scientific Evidence. J Behav Med. 2007 Feb 1;30(1):77–94. doi: 10.1007/s10865-006-9085-0 17180640

[54] LeBlanc M, Beaulieu-Bonneau S, Mérette C, Savard J, Ivers H, Morin CM. Psychological and health-related quality of life factors associated with insomnia in a population-based sample. Journal of Psychosomatic Research. 2007 Aug 1;63(2):157–66. doi: 10.1016/j.jpsychores.2007.03.004 17662752

