# Correction: Cognitive-behavioural pathways from pain to poor sleep quality and emotional distress in the general population: The indirect effect of sleep-related anxiety and sleep hygiene

**DOI:** 10.1371/journal.pone.0263758

**Published:** 2022-02-03

**Authors:** The PLOS ONE Staff

## Notice of Republication

This article was republished on January 25, 2022, to remove a Supporting Information file that was incorrectly included in the originally published article. The publisher apologizes for the error. Please download this article again to view the correct version.
