# Supplementary material for: Cognitive-behavioural pathways from pain to poor sleep quality and emotional distress in the general population: The indirect effect of sleep-related anxiety and sleep hygiene
Source: PLoS One. 2022 Jan 21;17(1):e0260614. doi: 10.1371/journal.pone.0260614 (PMC8782309; doi:10.1371/journal.pone.0260614)
Supplement: S1 Table — (DOCX) [file pone.0260614.s001.docx]

tableS1. Body map area

| Body map area | % |  |
| --- | --- | --- |
| Area 1 | 2.24 | Head |
| Area 22 | 16.42 | Head |
| Area 11 | 0.75 | Upper back |
| Area 12 | 1.49 | Upper back |
| Area 13 | 8.21 | Spine and low back |
| Area 2 | 19.4 | Spine and low back |
| Area 14a | 4.48 | Hip/thigh |
| Area 18a | 2.24 | Hip/thigh |
| Area 18b | 1.49 | Hip/thigh |
| Area 35 | 2.99 | Hip/thigh |
| Area 39 | 1.49 | Hip/thigh |
| Area 16 | 0.75 | Knee, lower leg, ankle or foot |
| Area 17 | 1.49 | Knee, lower leg, ankle or foot |
| Area 21 | 2.24 | Knee, lower leg, ankle or foot |
| Area 36 | 1.49 | Knee, lower leg, ankle or foot |
| Area 37 | 0.75 | Knee, lower leg, ankle or foot |
| Area 38 | 2.24 | Knee, lower leg, ankle or foot |
| Area 40 | 3.73 | Knee, lower leg, ankle or foot |
| Area 42 | 4.48 | Knee, lower leg, ankle or foot |
| Area 23 | 0.75 | Neck |
| Area 24 | 1.49 | Shoulder |
| Area 3 | 2.24 | Shoulder |
| Area 7 | 1.49 | Shoulder |
| Area 26 | 0.75 | Forearm, wrist and hand |
| Area 31 | 0.75 | Forearm, wrist and hand |
| Area 9 | 0.75 | Forearm, wrist and hand |
| Area 33 | 1.49 | Chest |
| Area 34 | 8.21 | Abdomen |
| Missing | 3.73 | Missing |
