# Supplementary material for: Cognitive-behavioural pathways from pain to poor sleep quality and emotional distress in the general population: The indirect effect of sleep-related anxiety and sleep hygiene
Source: PLoS One. 2022 Jan 21;17(1):e0260614. doi: 10.1371/journal.pone.0260614 (PMC8782309; doi:10.1371/journal.pone.0260614)
Supplement: S2 Table — (DOCX) [file pone.0260614.s002.docx]

tableS2. Pain locations

| Body part | % |
| --- | --- |
| Spine and low back | 28% |
| Head | 19% |
| Knee, lower leg, ankle or foot | 17% |
| Hip/thigh | 13% |
| Abdomen | 8% |
| Shoulder | 5% |
| Upper back | 2% |
| Forearm, wrist and hand | 2% |
| Neck | 1% |
| Chest | 1% |
| Missing | 4% |
